# Graves’ disease and the risk of Parkinson’s disease: a Korean population-based study

**DOI:** 10.1093/braincomms/fcac014

**Published:** 2022-02-07

**Authors:** Yoon Young Cho, Bongseong Kim, Dong Wook Shin, Jinyoung Youn, Ji Oh Mok, Chul-Hee Kim, Sun Wook Kim, Jae Hoon Chung, Kyungdo Han, Tae Hyuk Kim

**Affiliations:** Division of Endocrinology and Metabolism, Department of Medicine, Soonchunhyang University Bucheon Hospital, Bucheon, Korea; Department of Statistics and Actuarial Science, Soongsil University, Seoul, Korea; Supportive Care Center/Department of Family Medicine, Samsung Medical Center, Sungkyunkwan University School of Medicine, Seoul, Korea; Department of Clinical Research Design & Evaluation, Samsung Advanced Institute for Health Science & Technology, Sungkyunkwan University, Seoul, Korea; Department of Neurology, Samsung Medical Center, Sungkyunkwan University School of Medicine, Seoul, Korea; Neuroscience Center, Samsung Medical Center, Seoul, Korea; Division of Endocrinology and Metabolism, Department of Medicine, Soonchunhyang University Bucheon Hospital, Bucheon, Korea; Division of Endocrinology and Metabolism, Department of Medicine, Soonchunhyang University Bucheon Hospital, Bucheon, Korea; Division of Endocrinology and Metabolism, Department of Medicine, Thyroid Center, Samsung Medical Center, Sungkyunkwan University School of Medicine, Seoul, Korea; Division of Endocrinology and Metabolism, Department of Medicine, Thyroid Center, Samsung Medical Center, Sungkyunkwan University School of Medicine, Seoul, Korea; Department of Statistics and Actuarial Science, Soongsil University, Seoul, Korea; Division of Endocrinology and Metabolism, Department of Medicine, Thyroid Center, Samsung Medical Center, Sungkyunkwan University School of Medicine, Seoul, Korea

**Keywords:** Graves’ disease, Parkinson’s disease, population-based study, Korea

## Abstract

Two European cohort studies have suggested that Graves’ disease is associated with the development of Parkinson’s disease, although the results were limited and controversial. We evaluated whether patients with Graves’ disease had an increased risk of developing Parkinson’s disease according to treatment modality. We included 65 380 Graves’ disease patients and 326 900 healthy controls matched according to age and sex, using the Korean National Health Insurance database. The primary outcome was the incidences of Parkinson’s disease amongst Graves’ disease patients and controls. Subgroup analyses of Graves’ disease patients were performed according to anti-thyroid drug treatment, radioactive iodine therapy and surgery. The cumulative dose and duration values of anti-thyroid drug were calculated for each patient and categorized into highest, middle and lowest tertiles. Amongst 65 380 Graves’ disease patients, 301 Parkinson’s disease cases were diagnosed during 453 654 person-years of follow-up. Relative to the controls, and regardless of age, sex or comorbidities, the Graves’ disease patients had a 33% higher risk of developing Parkinson’s disease (hazard ratio: 1.33, 95% confidence interval: 1.17–1.51). Most Graves’ disease patients (96%) had received medical therapy, and increased risks of Parkinson’s disease were observed in the various subgroups for cumulative dose and treatment duration. This study revealed that Graves’ disease was an independent risk factor for developing Parkinson’s disease, and that the risk remained elevated regardless of demographic factors or treatment duration/dosage of the anti-thyroid drug. Clinicians should be aware that Graves’ disease patients have an increased risk of developing Parkinson’s disease, even though Graves’ disease patients are often relatively young.

## Teaser

The dopaminergic system interacts with thyroid hormones, however, evidence regarding Graves’ disease and Parkinson’s disease is scarce. This is the first epidemiological study on the effect of Graves’ disease on incident Parkinson’s disease and Kim *et al.* demonstrate that Graves’ disease is an independent risk factor for developing Parkinson’s disease.

## Graphical Abstract

**Graphical Abstract:**
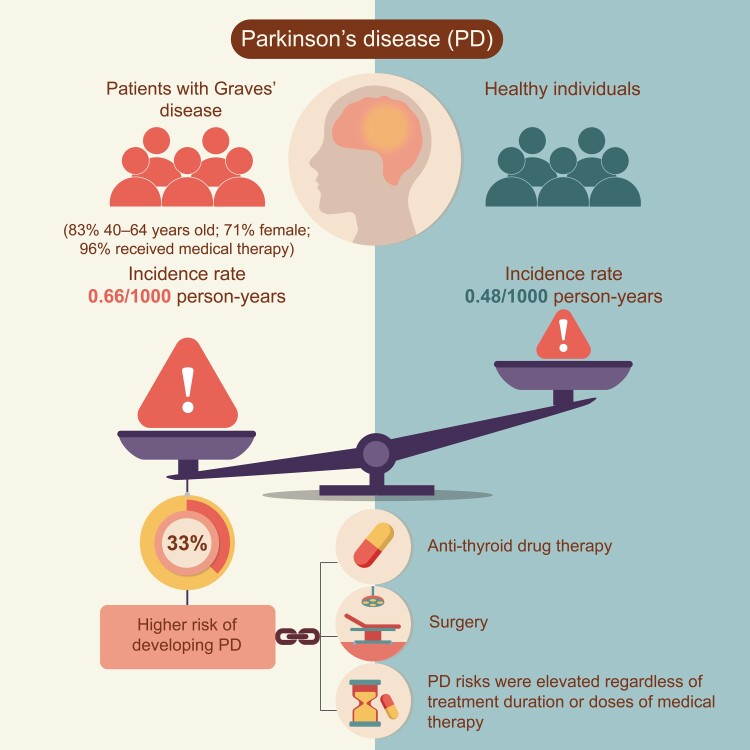

## Introduction

After Alzheimer’s disease, Parkinson’s disease is the second most common neurodegenerative disease. The incidence of Parkinson’s disease increases with age, affecting 1% of people who are >60 years old.^1^ This disease is characterized by progressive motor dysfunction, which can involve bradykinesia, resting tremors, rigidity and postural instability, as well as non-motor symptoms, such as sleep disturbance, depression, constipation and dysautonomic symptoms.^2^ Early-stage Parkinson’s disease leads to degeneration of dopaminergic neurons in the pars compacta of the substantia nigra, and loss of dopaminergic neurons is the most common post-mortem finding in brains that are affected by Parkinson’s disease.^3^

The dopaminergic system interacts with the hypothalamic–pituitary–thyroid (HPT) axis, with dopamine stimulating the production of thyrotropin-releasing hormone (TRH) and inhibiting the production of thyroid-stimulating hormone (TSH) and thyroid hormone. The HPT axis also regulates dopamine release. There is epidemiological evidence that thyroid diseases are associated with Parkinson’s disease,^4–6^ and similar genes contribute to both diseases,^7–10^ which may suggest that they share a common pathophysiological mechanism. Graves’ disease is an autoimmune thyroid disease that can lead to disturbances in dopamine transmission and the loss of dopaminergic neurons.^11,12^ However, we are only aware of two studies that have investigated the association between Graves’ disease and the subsequent development of Parkinson’s disease. Li *et al.*^5^ evaluated a Swedish nationwide database and reported that, relative to the healthy population, patients with Graves’ disease had an increased risk of developing Parkinson’s disease [standardized incidence ratio (SIR): 1.63, 95% confidence interval (CI): 1.39–1.90]. Furthermore, an even higher risk of developing Parkinson’s disease was observed amongst patients with Graves’ disease who were <65 years old (SIR: 1.72, 95% CI: 1.02–2.73), although the risk of Parkinson’s disease was not significantly increased at 5 years after the Graves’ disease diagnosis. Rugbjerg *et al.*^6^ performed a Danish population-based case–control study and reported that women with Graves’ disease had an increased risk of Parkinson’s disease (odds ratio [OR]: 2.1, 95% CI: 1.3–3.5), although the development of Parkinson’s disease was not associated with any other autoimmune diseases. However, both of these European cohort studies^5,6^ investigated the relationship between >30 autoimmune diseases and subsequent Parkinson’s disease, and could not support a detailed analysis of the association between Graves’ disease and Parkinson’s disease. Therefore, the present study evaluated whether Korean patients with Graves’ disease had an increased risk of developing Parkinson’s disease, using nationally representative data from the National Health Information Database (NHID), as well as whether the risk of developing Parkinson’s disease was associated with the Graves’ disease treatment modality and/or the cumulative dose and duration of anti-thyroid drug (ATD) treatment.

## Methods

### Data source

Data were retrieved from the Korean NHID, which is maintained by the National Health Insurance Service (NHIS). The NHIS is the only public medical insurance system in Korea and covers ∼97% of the Korean population, with the other 3% being covered by Medicaid. The NHID contains nationally representative data regarding healthcare utilization, health screening, sociodemographic variables and mortality, with healthcare utilization and drug prescriptions linked to International Classification of Diseases, 10th revision (ICD-10) diagnostic codes.^13^

### Study population

We identified 103 932 individuals who were diagnosed with Graves’ disease between January 2009 and December 2014 using the ICD-10 code for hyperthyroidism (E05). Patients with Graves’ disease were grouped according to whether they had received ATDs for ≥60 consecutive days (the ATD group), had undergone radioactive iodine therapy (the RAIT group) or had undergone thyroid surgery (the surgery group). Patients who underwent RAIT or surgery for Graves’ disease were assigned to the RAIT group or surgery group, regardless of whether they had received ATDs. The definition for detecting Graves’ disease cases used in this study has been used in the previous epidemiologic studies for Graves’ disease in Korea.^14,15^ Patients with thyroid carcinoma were excluded (ICD-10 code: C73.9). To exclude pre-existing Graves’ disease or Parkinson’s disease, a washout period was applied from January 2006 to study enrolment. Furthermore, as Parkinson’s disease is uncommon amongst individuals who are <40 years old,^16^ we excluded patients who were <40 years old, patients who were diagnosed with Parkinson’s disease during the washout period, or patients with missing data. Thus, the analysis included 65 380 Graves’ disease cases, who were matched with 326 900 controls (1:5 ratio) according to age and sex. The study period ended in December 2018.

The retrospective study protocol was approved by the Samsung Medical Center’s institutional review board (2019-01-034). The requirement for informed patient consent was waived based on the use of publicly available and de-identified patient data. All procedures for this study complied with the relevant patient confidentiality guidelines.

### Clinical variables

Comorbidities, including diabetes, hypertension and dyslipidaemia, were defined based on ICD-10 codes for diagnoses and drug prescriptions. Household income was classified into quartiles (Q1–Q4) and absolute poverty. The cumulative ATD dose was determined for each patient and classified as <4953 mg (lowest tertile), 4953–18 700 mg (middle tertile) and >18 700 mg (highest tertile). The ATD treatment duration was also determined for each patient and classified as <12 months (lowest tertile), 12–35 months (middle tertile) and >35 months (highest tertile).

### Identification of new Parkinson’s disease cases

The NHIS includes a registration programme for rare intractable diseases (RIDs), which include Parkinson’s disease. This programme was launched in 2006 and offers a copayment rate reduction of up to 10% (versus 20–30% for other common diseases). The patient is eligible for this RID programme after their physician submits a diagnosis certification. For cases of Parkinson’s disease, the certification is generally written by neurologists or primary care physicians who have examined the patient. New Parkinson’s disease onset was defined as a claim with the ICD-10 code for Parkinson’s disease (G20) and with a special RID registration code that indicates Parkinson’s disease (V124). This definition has been used in previous Korean epidemiological studies of Parkinson’s disease.^16–19^

### Statistical analysis

Categorical baseline characteristics were compared using the χ^2^ test. Conventional Cox proportional hazard regression analyses were performed to evaluate the association between Graves’ disease and incident Parkinson’s disease. The incidence rate (IR) was not adjusted in Model 1, whilst Model 2 was adjusted for age, sex, household income and comorbidities. The *P*-values for the interaction were calculated according to age, sex and comorbidities, which included diabetes, hypertension and dyslipidaemia. All statistical analyses were performed using SAS software (version 9.4; SAS Institute Inc., Cary, NC, USA).

### Data availability

The datasets generated and/or analyzed during the current study are available from the corresponding author on reasonable request.

## Results

### Demographic characteristics

The study included 65 380 Graves’ disease patients and 326 900 matched healthy controls. The mean age was 54 years, most subjects (83%) were <65 years old and 29% of subjects in both groups were men (Table 1). The Graves’ disease patients had higher prevalences of diabetes, hypertension and dyslipidaemia. In Graves’ disease patients, the majority of patients (95.8%) were treated with ATDs, followed by RAIT (3.4%) and surgery (0.8%). The RAIT group had a higher percentage of male, younger age and lower prevalences of diabetes and dyslipidaemia, compared to those of the ATD and surgery groups. The mean follow-up periods were 7.1 years in groups of Graves’ disease patients and controls.

**Table 1:** Baseline demographics of patients with Graves’ disease and controls

Variables	Controls (*n* = 326 900)	Graves’ disease (*n* = 65 380)	*P*-value	ATD (*n* = 62 615)	RAIT (*n* = 2237)	Surgery (*n* = 528)	*P*-value*
Male sex	95 175 (29%)	19 035 (29%)	1	18 148 (29%)	766 (34%)	121 (23%)	<0.0001
Mean age, years	54.2 ± 10.1	54.2 ± 10.1	1	54.3 ± 10.1	53.0 ± 9.8	54.0 ± 10.2	<0.0001
Age of ≥65 years	55 135 (17%)	11 027 (17%)	1	10 614 (17%)	320 (14%)	93 (18%)	0.004
Household income			<0.0001				0.009
Absolute poverty	19 615 (6%)	4226 (6%)		4045 (6%)	133 (6%)	48 (9%)	
First quartile	61 810 (19%)	11 932 (19%)		11 459 (18%)	389 (17%)	84 (16%)	
Second quartile	61 620 (19%)	11 689 (18%)		11 194 (18%)	384 (17%)	111 (21%)	
Third quartile	74 678 (23%)	14 700 (22%)		14 065 (23%)	506 (23%)	129 (24%)	
Fourth quartile	109 177 (33%)	22 833 (35%)		21 852 (35%)	825 (37%)	156 (30%)	
Diabetes	26 117 (8%)	8291 (13%)	<0.0001	7978 (13%)	234 (10%)	79 (15%)	0.002
Hypertension	78 202 (24%)	24 139 (37%)	<0.0001	22 932 (37%)	963 (43%)	244 (46%)	<0.0001
Dyslipidaemia	44 564 (14%)	10 518 (16%)	<0.0001	10 157 (16%)	273 (12%)	88 (17%)	<0.0001

ATD, anti-thyroid drug; RAIT, radioactive iodine therapy.

* *P*-value was calculated amongst three groups according to the treatment modality.

### Association of Graves’ disease and incident Parkinson’s disease

**Figure 1:**
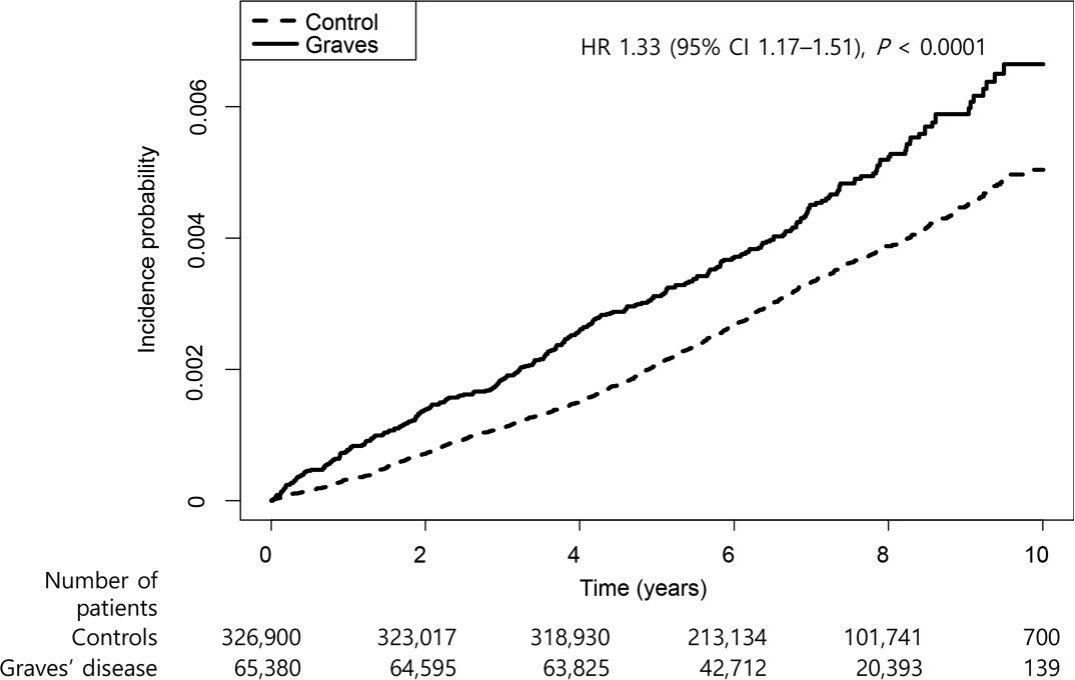
**Cumulative incidence of Parkinson’s disease amongst patients with Graves’ disease and controls.** Cox proportional hazard regression analyses were conducted and results were adjusted for age, sex, household income, diabetes, hypertension and dyslipidaemia.

Amongst the 65 380 Graves’ disease patients, 301 cases of incident Parkinson’s disease were diagnosed during 453 654 person-years (PYs) of follow-up. Relative to the controls, the Graves’ disease patients had a 37% higher risk of developing Parkinson’s disease [hazard ratio (HR): 1.37, 95% CI: 1.21–1.56]. Furthermore, the Graves’ disease patients still had an increased risk of developing Parkinson’s disease after adjusting for age, sex, household income and comorbidities (HR: 1.33, 95% CI: 1.17–1.51) (Fig. 1).

Subgroup analyses were performed according to age, sex and comorbidities, which generally revealed significantly higher risks of Parkinson’s disease amongst patients with Graves’ disease. However, non-significantly higher risks of Parkinson’s disease development were observed amongst Graves’ disease patients with diabetes (HR: 1.10, 95% CI: 0.83–1.46) and Graves’ disease patients without hypertension (HR: 1.20, 95% CI: 0.97–1.47) (Table 2).

**Table 2:** Subgroup analyses of Parkinson’s disease risk amongst patients with Graves’ disease and controls

Subgroup	Graves’ disease	*n*	Parkinson’s disease	PYs	IR per 1000 PYs	Hazard ratio (95% CI)^a^	*P* for interaction
40–64 years	No	271 765	417	1 911 919	0.22	Reference	0.92
	Yes	54 353	115	382 998	0.30	1.33 (1.08–1.64)	
≥65 years	No	55 135	680	354 584	1.92	Reference	
	Yes	11 027	186	70 655	2.63	1.34 (1.13–1.57)	
Male	No	95 175	285	653 355	0.44	Reference	0.53
	Yes	19 035	84	131 059	0.64	1.43 (1.12–1.83)	
Female	No	231 725	812	1 613 148	0.50	Reference	
	Yes	46 345	217	322 594	0.67	1.29 (1.11–1.50)	
No diabetes	No	300 783	897	2 095 931	0.43	Reference	0.15
	Yes	57 089	236	398 425	0.59	1.40 (1.21–1.61)	
Diabetes	No	26 117	200	170 572	1.17	Reference	
	Yes	8291	65	55 229	1.18	1.10 (0.83–1.46)	
No hypertension	No	248 698	582	1 741 618	0.33	Reference	0.18
	Yes	41 241	107	288 571	0.37	1.20 (0.97–1.47)	
Hypertension	No	78 202	515	524 885	0.98	Reference	
	Yes	24 139	194	165 083	1.18	1.41 (1.19–1.67)	
No dyslipidaemia	No	282 336	803	1 972 689	0.41	Reference	0.15
	Yes	54 862	193	384 279	0.50	1.22 (1.04–1.43)	
Dyslipidaemia	No	44 564	294	293 814	1.00	Reference	
	Yes	10 518	108	69 375	1.56	1.55 (1.24–1.93)	

PYs, person-years; IR, incidence rate.

^a^ Adjusted for age, sex, household income, diabetes, hypertension and dyslipidaemia.

### Association between Graves’ disease and incident Parkinson’s disease according to treatment modality

Relative to the controls, the ATD group had a 31% higher risk of developing Parkinson’s disease (HR: 1.31, 95% CI: 1.15–1.50) and the surgery group had a 208% higher risk of developing Parkinson’s disease (HR: 3.08, 95% CI: 1.28–7.43). However, the RAIT group did not have a significantly higher risk of developing Parkinson’s disease (HR: 1.34, 95% CI: 0.70–2.59) (Table 3). The risk of developing Parkinson’s disease did not vary according to whether the patients achieved Graves’ disease remission after undergoing RAIT (data not shown).

**Table 3:** Risks of Parkinson’s disease amongst patients with Graves’ disease according to treatment modality

	*n*	Parkinson’s disease	PYs	IR per 1000 PYs	Model 1^a^	Model 2^b^
Controls	326 900	1097	2 266 503	0.48	Reference	Reference
Graves’ disease	65 380	301	463 654	0.66	1.37 (1.21–1.56)	1.33 (1.17–1.51)
Treatment modality						
ATD	62 615	287	434 451	0.66	1.37 (1.20–1.55)	1.31 (1.15–1.50)
RAIT	2237	9	15 634	0.58	1.19 (0.62–2.29)	1.34 (0.70–2.59)
Surgery	528	5	3569	1.40	2.91 (1.21–6.99)	3.08 (1.28–7.43)

PYs, person-years; IR, incidence rate; ATD, anti-thyroid drug; RAIT, radioactive iodine therapy.

^a^ Not adjusted.

^b^ Adjusted for age, sex, household income, diabetes, hypertension and dyslipidaemia.

### Subgroup analyses of associations between Graves’ disease treatment modalities and incident Parkinson’s disease

Subgroup analyses were performed according to age, sex, comorbidities and treatments for Graves’ disease. Most subgroups of Graves’ disease patients had similar risks of developing Parkinson’s disease, regardless of demographic characteristics or treatment modality (non-significant *P*-values for interaction). In particular, Graves’ disease patients who were 40–64 years old (*n *= 54 353, 83% of the Graves’ disease population) had increased risks of developing Parkinson’s disease, regardless of whether their treatment had involved ATDs (HR: 1.27, 95% CI: 1.03–1.58), RAIT (HR: 2.47, 95% CI: 1.17–5.21) or surgery (HR: 4.34, 95% CI: 1.39–13.51) (Table 4). Moreover, subgroup analyses according to cumulative ATD dose and treatment duration revealed relatively consistent increased risks of developing Parkinson’s disease, relative to the controls. The one exception was a non-significant difference in the group with the longest ATD treatment duration (HR: 1.03, 95% CI: 0.82–1.29) (Table 5).

**Table 4:** Subgroup analyses of Parkinson’s disease risk amongst patients with Graves’ disease and controls according to treatment modality

Subgroup	Treatment	*n*	Parkinson’s disease	PYs	IR per 1000 PYs	Hazard ratio (95% CI)^a^	*P* for interaction
40–64 years	Controls	271 765	417	1 911 919	0.22	Reference	0.20
	ATD	52 001	105	366 430	0.29	1.27 (1.03–1.58)	
	RAIT	1917	7	13 566	0.52	2.47 (1.17–5.21)	
	Surgery	435	3	3002	0.99	4.34 (1.39–13.51)	
≥65 years	Controls	55 135	680	354 584	1.92	Reference	
	ATD	10 614	182	68 021	2.68	1.35 (1.15–1.60)	
	RAIT	320	2	2067	0.97	0.51 (0.13–2.05)	
	Surgery	93	2	567	3.53	1.85 (0.49–7.86)	
Male sex	Controls	95 175	285	653 355	0.44	Reference	0.51
	ATD	18 148	81	124 883	0.65	1.43 (1.12–1.84)	
	RAIT	766	1	5368	0.19	0.58 (0.08–4.15)	
	Surgery	121	2	808	2.48	5.25 (1.31–21.12)	
Female sex	Controls	231 725	812	1 613 148	0.50	Reference	
	ATD	44 467	206	309 567	0.67	1.27 (1.09–1.48)	
	RAIT	1471	8	10 266	0.78	1.62 (0.81–3.26)	
	Surgery	407	3	2761	1.09	2.38 (0.77–7.39)	
No diabetes	Controls	300 783	897	2 095 931	0.43	Reference	0.64
	ATD	54 637	224	381 315	0.59	1.38 (1.19–1.60)	
	RAIT	2003	7	14 036	0.50	1.39 (0.66–2.90)	
	Surgery	449	5	3074	1.63	4.20 (1.74–10.12)	
Diabetes	Controls	26 117	200	170 572	1.17	Reference	
	ATD	7978	63	53 136	1.19	1.11 (0.83–1.47)	
	RAIT	234	2	1597	1.25	1.23 (0.31–4.96)	
	Surgery	79	0	495	0	–	
No hypertension	Controls	248 698	582	1 741 618	0.33	Reference	0.28
	ATD	39 683	102	277 662	0.37	1.18 (0.96–1.46)	
	RAIT	1274	2	8964	0.22	0.82 (0.20–3.28)	
	Surgery	284	3	1944	1.54	5.20 (1.68–16.16)	
Hypertension	Controls	78 202	515	524 885	0.98	Reference	
	ATD	22 932	185	156 789	1.18	1.40 (1.19–1.66)	
	RAIT	963	7	6669	1.05	1.60 (0.76–3.38)	
	Surgery	224	2	1624	1.23	1.86 (0.46–7.46)	
No dyslipidaemia	Controls	282 336	803	1 972 689	0.41	Reference	0.37
	ATD	52 458	182	367 414	0.50	1.20 (1.02–1.41)	
	RAIT	1964	7	13 859	0.51	1.40 (0.67–2.95)	
	Surgery	440	4	3005	1.33	3.62 (1.35–9.66)	
Dyslipidaemia	Controls	44 564	294	293 814	1.00	Reference	
	ATD	10 157	105	67 037	1.57	1.56 (1.25–1.95)	
	RAIT	273	2	1775	1.13	1.11 (0.28–4.45)	
	Surgery	88	1	563	1.77	1.97 (0.28–14.06)	

PYs, person-years; IR, incidence rate; ATD, anti-thyroid drug; RAIT, radioactive iodine therapy.

^a^ Adjusted for age, sex, household income, diabetes, hypertension and dyslipidaemia.

**Table 5:** Risks of Parkinson’s disease amongst patients with Graves’ disease according to cumulative ATD dose and treatment duration

	*n*	Parkinson’s disease	PYs	IR per 1000 PYs	Model 1^a^	Model 2^b^
Controls	326 900	1097	2 266 503	0.48	Reference	Reference
ATD	62 615	287	434 451	0.66	1.37 (1.20–1.55)	1.31 (1.15–1.50)
Cumulative dose						
Lowest	20 871	96	136 217	0.70	1.48 (1.20–1.83)	1.33 (1.08–1.60)
Middle	20 875	91	142 299	0.64	1.33 (1.07–1.65)	1.31 (1.06–1.62)
Highest	20 869	100	155 935	0.64	1.30 (1.06–1.60)	1.31 (1.06–1.61)
Cumulative duration						
Lowest	20 889	109	142 228	0.77	1.59 (1.30–1.93)	1.49 (1.23–1.82)
Middle	20 857	95	143 451	0.66	1.37 (1.11–1.69)	1.46 (1.19–1.81)
Highest	20 869	83	148 772	0.56	1.15 (0.92–1.43)	1.03 (0.82–1.29)

PYs, person-years; IR, incidence rate; ATD, anti-thyroid drug.

Cumulative ATD doses were <4953 mg (lowest), 4953–18 700 mg (middle) and >18 700 mg (highest).

Cumulative ATD treatment durations were <12 months (lowest), 12–35 months (middle) and >35 months (highest).

^a^ Not adjusted.

^b^ Adjusted for age, sex, household income, diabetes, hypertension and dyslipidaemia.

## Discussion

The present study revealed that Korean Graves’ disease patients had a 33% higher risk of developing Parkinson’s disease, relative to age- and sex-matched healthy controls, based on nationally representative population-based data. During 453 654 PYs of follow-up for 65 380 Graves’ disease patients, a total of 301 incident Parkinson’s disease cases were identified, which corresponded to an IR of 0.66 cases per 1000 PYs (versus 0.48 cases per 1000 PYs amongst the healthy controls). Furthermore, the increased risk of developing Parkinson’s disease amongst Graves’ disease patients remained consistent in various subgroups that were created according to age, sex or comorbidities. Moreover, the Graves’ disease treatment modality, except RAIT and clinical courses did not appear to influence the risk of developing Parkinson’s disease, which suggests that Graves’ disease itself is a risk factor for Parkinson’s disease.

The most important role of dopamine involves the inhibition of prolactin synthesis, whilst TRH stimulates prolactin synthesis in the absence of dopamine.^20^ However, the dopaminergic system and HPT axis are also interconnected outside the pituitary gland. For example, dopamine and D2 dopamine receptors mediate the direct stimulation of TRH in the striatum. Dopamine and TRH also upregulate each other, whilst dopamine inhibits the production of TSH and thyroid hormones. Furthermore, imbalances in thyroid hormones lead to dopamine degradation, downregulation of dopamine hydroxylase and alterations in dopaminergic receptor sensitivity.^12^ Dopamine signalling and the HPT axis are linked with sleep.^21,22^ Dopamine promotes sleep and dopamine deficiency leads to the increase in TSH and thyroid hormone secretion.^21,22^ A hypothalamic imbalance between neuro-excitatory thyroid hormones and neuro-inhibitory dopamine secretion has been proposed to be the causative mechanism of restless leg syndrome.^12^ Oxidative stress provides important contributions to dopamine neuron loss and Parkinson’s disease progression^23^ and thyroid dysregulation affects oxidative stress because thyroid hormones affect both oxidant and anti-oxidant activities.^24^ As the common environmental factor, vitamin D deficiency has been suggested as a possible risk factor for both Graves’ disease and Parkinson’s disease^25–27^; however, there are controversies for the causal relationship between vitamin D deficiency and Graves’ disease. In addition, there is evidence regarding common abnormalities that increase susceptibility to both Parkinson’s disease and thyroid disease, which may involve *RASD2*,^7^  *WSB1*,^8^  *MAPT*^10^ and NOX/DUOX.^9^

Two European cohort studies have provided epidemiological evidence that Graves’ disease may be associated with an increased risk of developing Parkinson’s disease. Li *et al.*^5^ evaluated 34 735 Graves’ disease/hyperthyroidism patients and reported a 63% higher risk of developing Parkinson’s disease, which was maintained when they excluded the first year after the Graves’ disease diagnosis (SIR: 1.42, 95% CI: 1.19–1.68). However, the risk of Parkinson’s disease was not significantly increased at 5 years after the index date (SIR: 1.17, 95% CI: 0.94–1.44), which the authors attributed to lead-time bias and earlier diagnosis of Parkinson’s disease during the first few years after the Graves’ disease diagnosis. In contrast, the present study revealed that the cumulative incidence of Parkinson’s disease increased steadily, even at 5 years after the Graves’ disease diagnosis (Fig. 1). This result is likely reliable, as the diagnosis of Parkinson’s disease is registered and supervised under the Korean RID programme, which ensures that the diagnoses are accurate.^16,17^ In addition, we used age- and sex-matched controls, with correction for comorbidities and household income, which ensures a clearer analysis of the association between two diseases, relative to the SIR values that were reported by Li *et al.*^5^ Rugbjerg *et al.*^6^ also reported that Graves’ disease patients had an increased risk of Parkinson’s disease (OR: 2.1), although they did not further evaluate the association between Graves’ disease and Parkinson’s disease. Whilst those studies of Swedish and Danish cohorts^5,6^ were well designed and used validated data, they evaluated a broad range of >30 autoimmune diseases and were not sufficiently powered to specifically analyze the association between Graves’ disease and Parkinson’s disease.

Older age and a family history of Parkinson’s disease are the most important risk factors for developing Parkinson’s disease,^28^ although there is controversy regarding other risk factors. We found that the incidence of Parkinson’s disease was high amongst Graves’ disease patients who were ≥65 years old (IR: 2.63 per 1000 PYs), even amongst the healthy controls (IR: 1.92 per 1000 PYs), relative to amongst controls who were 40–64 years old (IR: 0.22 per 1000 PYs), as with the fact that older age is a most critical risk factor for developing Parkinson’s disease. Of note, elderly people (≥65 years old) with Graves’ disease had a 34% higher risk of Parkinson’s disease, even after statistical correction for their comorbidities that might be contributing factors for Parkinson’s disease. In this elderly Graves’ disease population, only ATD group showed the elevated risk (HR: 1.35, 95% CI: 1.15–1.60) for Parkinson’s disease, whereas the RAIT (HR: 0.51, 95% CI: 0.13–2.05) and surgery groups (HR: 1.85, 95% CI: 0.49–7.86) did not. However, non-significant results may be derived from the small number of Parkinson’s disease events in the RAIT (two Parkinson’s disease events in 320 patients) and surgery groups (two Parkinson’s disease events in 93 patients); thus, it is cautious to conclude that RAIT and surgical treatment are better than medical treatment for preventing incident Parkinson’s disease in this elderly Graves’ disease population.

Most of the Graves’ disease patients (83%) were <65 years old and had an increased risk of Parkinson’s disease (HR: 1.33), although the absolute increase was not large (IR: 0.3 per 1000 PYs), which agrees with the SIR of 1.72 reported by Li *et al.*^5^ amongst patients who were <65 years old. Furthermore, control subjects with comorbidities (diabetes, hypertension or dyslipidaemia) tended to have a higher incidence of Parkinson’s disease, relative to control subjects without comorbidities (Table 2). This also agrees with a previous report that the risk of Parkinson’s disease is elevated amongst patients with diabetes.^17,29^ Nevertheless, the Graves’ disease patients had elevated risks of Parkinson’s disease, regardless of their age, sex or comorbidities, which suggests that Graves’ disease may be an independent risk factor for developing Parkinson’s disease.

To the best of our knowledge, ours is the first study to evaluate whether the risk of Parkinson’s disease was related to the modality used to treat Graves’ disease. Treatment groups with ATD and surgery had increased risks of developing Parkinson’s disease, whereas, the risk was not elevated in the RAIT group. Interestingly, although <1% of the Graves’ disease patients had undergone surgery, they had a substantially higher risk of developing Parkinson’s disease (versus the ATD group), although we do not have a clear explanation for this finding. One assumption is that baseline demographics, including a higher percentage of female, absolute property, diabetes and hypertension in the surgery group (Table 1) might be linked with the higher risk for incident Parkinson’s disease, compared to other two groups, in spite of the statistical correction. In contrast, the RAIT group had a relatively low female incidence, younger mean age, the better economic status, lower rates of diabetes and dyslipidaemia compared to those of the ATD and surgery groups, which might be favourable factors for incident Parkinson’s disease. Although there has been no evidence regarding incident Parkinson’s disease by treatment modality for Graves’ disease, some literatures have reported that Graves’ disease patients achieving remission after RAIT had the lower risk of cardiovascular morbidity and mortality.^30,31^ Based on our results, RAIT might be a better treatment option for Graves’ disease regarding the incident risk of Parkinson’s disease, although further research with the larger population for RAIT is needed.

It is also interesting that the risks of Parkinson’s disease were similar between the subgroups that were classified according to cumulative ATD dose or ATD treatment duration, despite the fact that a higher cumulative dose or prolonged treatment would suggest the cases involved obstinate Graves’ disease. Therefore, although we had limited information regarding whether the Graves’ disease was persistent or in remission, it appears that Graves’ disease itself may be a potential risk factor for Parkinson’s disease.

The present study has several limitations. First, the diagnosis of hyperthyroidism was based on ICD-10 codes and information regarding the aetiology of hyperthyroidism was lacking. To exclude transient thyrotoxicosis, the definition of consecutive ATD prescription was also used for the ATD group. The aetiology of hyperthyroidism in an iodine-sufficient area, including Korea is known as Graves’ disease, and toxic adenoma accounts for <1% of the aetiology of hyperthyroidism in Korea.^32^ Therefore, the present results may be interpreted as the results of Graves’ disease. Second, we identified Parkinson’s disease cases using the RID database. An epidemiologic study for the incidence of Parkinson’s disease in Korea using the NHIS-RID database^16^ reported the gradual increase in the incidence of Parkinson’s disease from 23.2 to 27.8 per 100 000 (2010–15) in Korean population (male, 18.9–23.3 per 100 000 and female, 27.4–32.2 per 100 000). In our population, the incidence of Parkinson’s disease was 48 per 100 000 in controls and the incidence was somewhat higher than that of the study by Park *et al.*’s,^16^ although the direct comparison of the incidence of Parkinson’s disease between our study and the previous study by Park *et al.* seems to be inappropriate because controls in this study were matched with Graves’ disease population by age and sex. Female predominance and elderly population excluding individuals <40 years old in this study might affect the incidence of Parkinson’s disease. Third, the NHID does not include data regarding relevant clinical parameters, such as thyroid function or autoantibody titres (e.g. antibodies to TSH receptor), which precluded related analyses. Fourth, the mechanisms underlying the increased risk of Parkinson’s disease that is associated with Graves’ disease remain unclear. Fifth, ∼96% of the Korean Graves’ disease patients had received ATDs, which is substantially higher than the proportion of American patients that receive ATDs (∼60%).^33^ Thus, our findings might be influenced by the large proportion of patients who received medical treatment for Graves’ disease, and the findings might not be replicated in larger studies of patients who underwent RAIT or surgery for Graves’ disease. Nevertheless, the prevalences of Graves’ disease and Parkinson’s disease are both <1% in the general population, and a population-based database is likely needed to evaluate the relationship between Graves’ disease and Parkinson’s disease, despite the innate limitations of the NHID.

## Conclusions

The present study revealed that Korean patients with Graves’ disease had an increased risk of developing Parkinson’s disease, even at relatively young ages. Furthermore, the risk of Parkinson’s disease did not substantially change in subgroup analyses according to demographic characteristics or treatment duration/dosage of ATDs. Therefore, although the absolute incidence is low, we suggest that clinicians should remain aware of the possibility that Graves’ disease patients may develop Parkinson’s disease, even at relatively young ages.

## Abbreviations

ATD =: anti-thyroid drug
CI =: confidence interval
HPT =: hypothalamic–pituitary–thyroid
HR =: hazard ratio
ICD-10 =: International Classification of Diseases, 10th revision
IR =: incidence rate
PYs =: person-years
NHID =: the National Health Information Database
NHIS =: the National Health Insurance Service
RAIT =: radioactive iodine therapy
RIDs =: rare intractable diseases
SIR =: standardized incidence ratio
TRH =: thyrotropin-releasing hormone
TSH =: thyroid-stimulating hormone
